# Clinical and Microbiological Spectrum of Dermatophytosis From a Tertiary Care Institute

**DOI:** 10.7759/cureus.77523

**Published:** 2025-01-16

**Authors:** Sukanya Sudhaharan, Dipika Shaw, Sabaa Naaz, Savitha Kovida, Umabala Pamidimukkala

**Affiliations:** 1 Microbiology, Nizam's Institute of Medical Sciences, Hyderabad, IND; 2 Microbiology, Jawaharlal Nehru Medical College, Datta Meghe Institute of Higher Education and Research, Wardha, IND; 3 Microbiology, Nizam’s Institute of Medical Sciences, Hyderabad, IND

**Keywords:** chronic dermatophytosis, resistance, terbinafine, tinea, trichophyton

## Abstract

Introduction

There is an increasing incidence of recalcitrant dermatophytosis in India due to the irrational use of antifungals, inappropriate treatment, and in vitro resistance of the organism. The study aims to determine the clinic-mycological profile, antifungal susceptibility, and outcome of patients with dermatophytosis in our institute.

Methods

This was a retrospective observational study from January 2019 to December 2019. Detailed clinical data of all the enrolled patients were collected. Identification of the dermatophytes was performed by colony morphology and microscopy. Further, the preserved isolates were sent to the Post Graduate Institute of Medical Education & Research (PGIMER), Chandigarh for confirmation of identification and antifungal susceptibility testing.

Results

Of the 155 clinical suspected cases, growth of dermatophytes was observed in 55 (35.4%) of the cases. Tinea corporis (39/55, 70.9%) was the predominant clinical type. The duration of infection was less than six months in 22/55 (40%) of cases. Most patients were in the age group of 20-30 years. Diabetes mellitus was the predominant comorbid condition in 7/55 (12.7%) patients.

Previous therapy with topical and systemic antifungals was given in 32/55 (58.8%) of the patients. *Trichophyton mentagrophytes* complex (69%) was the predominant species complex isolated followed by *Microsporum gypseum *(10.9%)*.*

All *Trichophyton* isolates showed minimum inhibitory concentration (MIC) >1 µg/ml for fluconazole and griseofulvin. Two isolates showed mutation in the squalene epoxidase (SE) gene. In the present study, the patients were treated with both oral and topical antifungals.

Conclusion

*T. mentagrophytes* complex was the predominant species isolated and Tinea corporis was the commonest clinical presentation. Resistance to terbinafine, griseofulvin, and fluconazole has been noted.

## Introduction

Globally, superficial fungal infections account for 20-25% of the population, and dermatophytosis constitutes most of the infections [1]. Dermatophytes are keratinophilic fungi that affect the skin, hair and nails, causing a highly communicable disease [1,2]. Over the past few decades, many studies from India have reported the increased prevalence of dermatophytosis [3]. Moreover, the hot and humid climatic condition with increased temperature and excessive sweating promotes the growth of fungi [4,5]. The prevalence of the infection and the species isolated also vary in different parts of the country due to variations in lifestyle, socioeconomic conditions, and other risk factors in those areas. In India, fixed drug combination creams with steroid, and antifungal antibacterial are available in the market and irrational and improper usage of them without proper medical advice for years together can lead to resistance to antifungals, treatment failure and chronic recalcitrant dermatophytosis, which is of major concern [3, 6].

There are studies from different parts of the country [5-8] on dermatophytosis and its antifungal susceptibility. To date, there are no Clinical and Laboratory Standards Institute (CLSI) breakpoints for antifungal susceptibility testing in dermatophytes and there are few data related to the correlation between in-vivo and in-vitro susceptibility [9]. The present study is done to correlate the clinico-mycological profile and antifungal susceptibility of patients with dermatophytosis.

This article was previously presented as a poster at the 21st Congress of the International Society for the Human and Animal Mycology (ISHAM) 2022, held in New Delhi, India from 20th-24th September 2022.

## Materials and methods

This was a retrospective observational study from January 2019 to December 2019. There were a total of 155 clinically suspected cases of dermatophytosis, which were included in the study. Demographic data, clinical details, previous treatment history, and the prescribed treatment information were collected from the registers.

Inclusion criteria

All patients with clinically suspected dermatophytosis during that period were included in the study.

Exclusion criteria

Patients with other skin infections were excluded from the study.

Depending on the site of infection, clinical samples, i.e., skin scrapings, nail clippings, and hair samples, were collected. The clinical samples were subjected to direct microscopy, i.e., potassium-hydroxide (KOH) wet mount preparation (10% KOH for skin and hair; 40% KOH for the nail) and 0.1% calcofluor white stain for the presence of fungal elements. All the specimens were inoculated into a Sabouraud's dextrose agar with chloramphenicol, Sabouraud's dextrose agar with chloramphenicol and cycloheximide and incubated at 25°C for up to four weeks. Identification of the dermatophytes was performed by colony morphology and microscopy. Further, the preserved isolates were sent to the Post Graduate Institute of Medical Education & Research (PGIMER) Chandigarh for confirmation of identification and antifungal susceptibility testing (AFST). Antifungal susceptibility was done in Chandigarh as mentioned by Rudramurthy et al. [9]. Antifungal susceptibility testing was done for 47 isolates of Trichophyton species for eight drugs (Fluconazole, ketoconazole, clotrimazole, itraconazole, terbinafine, naftifine, ciclopirox olamine, and griseofulvin). Seventeen isolates were also tested for miconazole, luliconazole, voriconazole, sertaconazole, and amorolfine.

Fluconazole, voriconazole, ketoconazole, sertaconazole, clotrimazole, itraconazole, terbinafine, naftifine, amorolfine, ciclopirox olamine, and griseofulvin (from Sigma-Aldrich, Bengaluru, India) were used for antifungal susceptibility testing. Fluconazole was dissolved in distilled water, while all other antifungals were dissolved in dimethyl sulfoxide. The final concentrations of the antifungals tested ranged from 0.0625 to 32 μg/ml for fluconazole; 0.0312 to 16 for ketoconazole, clotrimazole, ciclopirox olamine, luliconazole, and naftifine; 0.0078 to 4 μg/ml for voriconazole, amorolfine, itraconazole, and sertaconazole; 0.0156 to 64 μg/ml for terbinafine and sertaconazole, and 0.25 to 128 μg/ml for griseofulvin [9]. The plates were incubated at 28°C, and readings were taken after four days. Endpoints of minimum inhibitory concentrations (MICs) for azoles, griseofulvin, and amorolfine were considered when they showed prominent inhibition of growth (approximately 80%) compared to that in growth control wells, while for terbinafine, naftifine, luliconazole, and ciclopirox olamine, 100% growth inhibition was noted. Four quality control strains, such as *T. mentagrophytes/ interdigitale *complex (NCCPF 800035), *Candida parapsilosis* (ATCC 22019), *Candida krusei* (ATCC 6258), and *Aspergillus flavus* (ATCC 204304), were used for AFST.

Statistical analysis

Descriptive statistics were used for analysis. Categorical data were described as frequencies with percentages. The data were entered into a Microsoft Excel sheet and analyzed using SPSS version 20.0 (IBM Corp., Armonk, NY, USA).

## Results

Of the 155 clinically diagnosed dermatophytosis cases, only 55 (35.4%) cases showed growth of dermatophytes. Further, no growth was observed in 84/155 (54.1%) cases, *Candida *in 10/55 (6.4), and growth of other non-dermatophytic molds like *Alternaria*, *Fusarium*, *Acremonium* was noted in 6/155 (3.8%). Both direct microscopy and culture were positive in 43/155 (27.7%) and only culture positive in 12/155 (7.75%) cases (Table 1).

**Table 1:** Microscopy and culture findings of patients with dermatophytosis

Microscopy and culture findings	Number of patients	Percentage
Microscopy positive, culture positive	43	27.7
Microscopy negative, culture positive	12	7.7

Among the 55 cases of dermatophytosis, the majority of the patients were in the age group of 20-30 years (Table 2).

**Table 2:** Age distribution of patients with dermatophytosis

S. No	Age in years	No. of patients
1	10-20	10
2	21-30	16
3	31-40	4
4	41-50	13
5	51-60	6
6	61-70	5
7	71-80	1

Male: Female ratio is 2.2:1. A total of 36/55 (65.4%) of the patients belong to middle socioeconomic status, and 19/55 to lower socioeconomic status (34.5%); 10/55 (19.2%) of the patients were students and 10/55 (19.2%) housewives. Others include auto drivers, mechanics, teachers, cashiers, etc. A total of 51/55 (92.7%) of the patients were from urban and 4/55 (7.2%) were from rural areas.

Sharing items with infected family members and hostel roommates was found in 12/55 (21.8%) patients. Excessive sweating was observed in 9/55 (16.3%) patients. The co-morbid conditions include diabetes mellitus in 7/55 (12.7%), hypertension in 6/55 (10.9%), steroid treatment in 3/55 (5.4%), post renal transplant in 1/55 (1.8%), and systemic lupus erythematosus (SLE) in 1/55 (1.8%) patients. The duration of infection was less than six months in 22/55 (40%) of cases and > 6 months in 33 (60%) of the cases.

The lesions were erythematous and pigmented in 5/55 (9%) and 2/55 (3.6%) patients respectively and 48/55 (87.2%) had scaly itching lesions. Patients sought medical attention at various durations of illness. 50/55 (90.9%) of the patients received treatment (including four patients with alternative medicine treatment) within 2-6 months of the onset of illness and 4/55 (7.2%) after 1-2 years of onset of illness. 1/55 (1.8%) of the patients with T. unguium received treatment after six years of onset.

*Trichophyton mentagrophytes* complex (n=38; 69%) was the predominant organism isolated, followed by *Microsporum gypseum* (n=6; 10.9%) (Table 3).

**Table 3:** Dermatophytes isolated from patients with dermatophytosis

Organism	No. of isolates	Percentage
T. mentagrophytes complex	38	69
T. rubrum	5	9
T. tonsurans	4	7
T. violaceum	1	1.8
M. gypseum	6	10.9
M. canis	1	1.8
Total	55	

**Figure 1:**
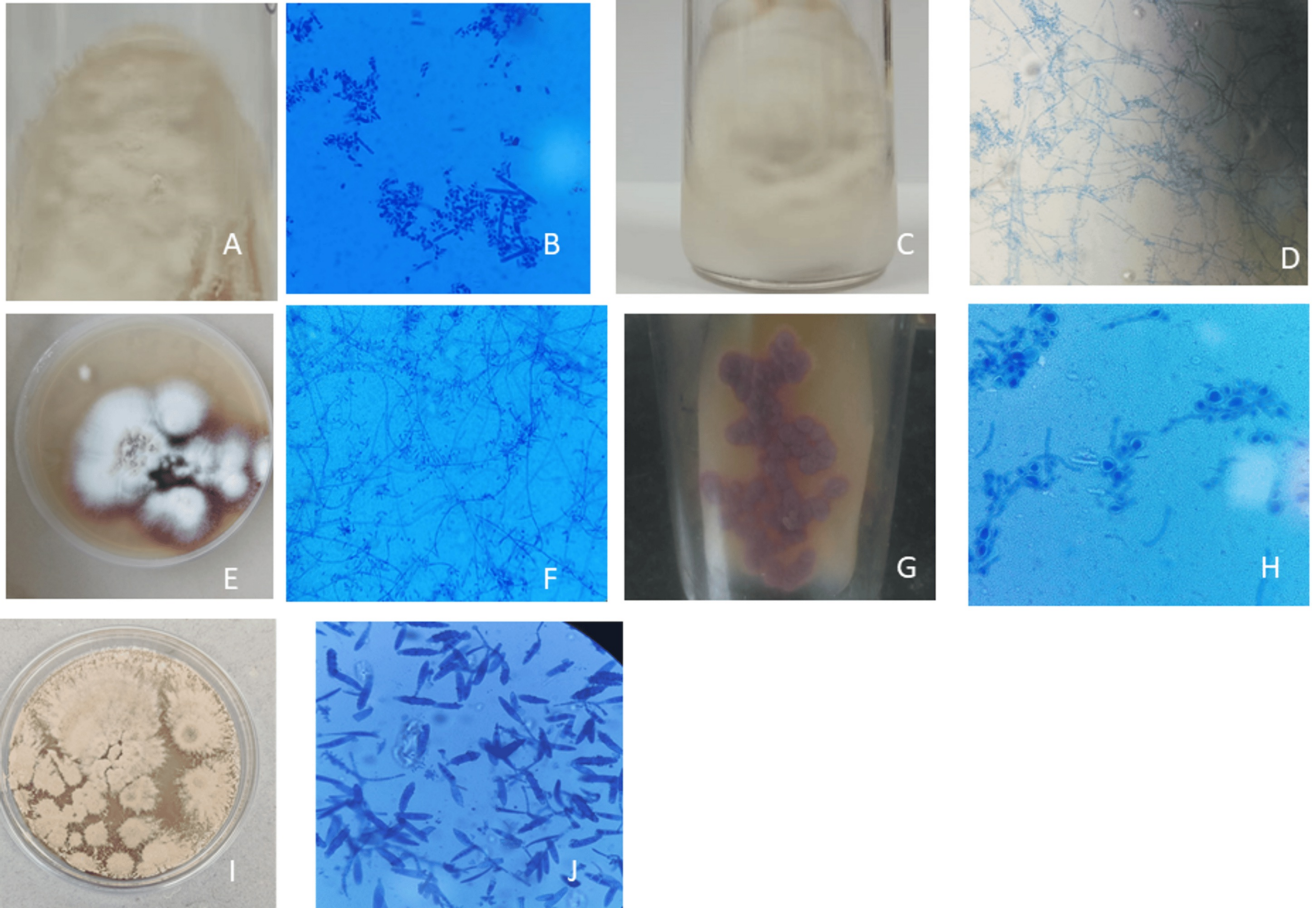
(1A) Culture of Trichophyton mentagrophytes complex on Sabouraud dextrose agar. (1B) Lactophenol cotton blue mount of Trichophyton mentagrophytes complex 400x magnification. (1C) Culture of Trichophyton tonsurans on Sabouraud dextrose agar. (1D) Lactophenol cotton blue mount of Trichophyton tonsurans 400x magnification. (1E) Culture of Trichophyton rubrum on Sabouraud dextrose agar. (1F) Lactophenol cotton blue mount of Trichophyton rubrum 400x magnification. (1G) Culture of Trichophyton violaceum on Sabouraud dextrose agar. (1H) Lactophenol cotton blue mount of Trichophyton violaceum 400x magnification. (1I) Culture of Microsporum gypseum on Sabouraud dextrose agar. (1J) Lactophenol cotton blue mount of Microsporum gypseum 400x magnification

Culture characteristics such as surface texture, topography and pigmentation are variable for each species and microscopic identification is based on the presence of microconidia and macroconidia. The culture and microscopy of different dermatophytes isolated are shown in Figure 1.

A mixed type of infection was noted in 10/55 (18.1%) patients. Tinea corporis was the predominant clinical presentation in 39/55 (70.9%) patients. Tinea corporis and tinea cruris (7/55 [12.7%]) were the most common coexistent forms of the disease. The site of infection to age and organism isolated is shown in Table 4 and Table 5, respectively.

**Table 4:** Age and sex-wise distribution with relation to clinical types of dermatophytosis Note: Mixed type Tinea corporis & capitis-1 Tinea corporis, cruris & manuum-1 Tinea corporis & cruris-7 Tinea corporis, cruris & faciei-1

Clinical types	Age group (years) (%)							Gender (%)		Total %
	10-20	20-30	30-40	40-50	50-60	60-70	70-80	M	F	
Tinea corporis	6	12	3	10	4	4	-	25	14	39 (70.9%)
Tinea capitis	1	-	1	-	-	-	-	2	0	2 (3.6%)
Tinea unguium	-	1	-	1	1	-	-	3	0	3 (5.4%)
Tinea faciei	-	1	-	-	-	-	-	1	0	1 (1.8%)
Mixed type	3	2	-	2	1	1	1	7	3	10 (18.1%)

**Table 5:** Dermatophytes isolated in relation to clinical types of dermatophytosis Note: Mixed type Tinea corporis & tinea capitis-1; Tinea corporis, cruris & manuum-1; Tinea corporis & tinea cruris-7; Tinea corporis, tinea cruris & tinea faciei-1

Clinical types	No. of patients	Total isolated (%)	Trichophyton rubrum	Trichophyton mentagrophytes complex	Trichophyton tonsurans	Trichophyton violaceum	Microsporum gypseum	Microsporum canis
Tinea corporis	39	70.9%	5	28	3	-	2	1
Tinea capitis	2	3.6%	-	-	1	1	-	-
Tinea unguium	3	5.4%	-	3	-	-	-	-
Tinea faciei	1	1.8%	-	1	-	-	-	-
Mixed type	10	18.1%	-	6	-	-	4	-

Antifungal susceptibility was done in PGIMER Chandigarh for *Trichophyton* spp. (n=47) (Table 6).

**Table 6:** The minimum inhibitory concentration (MIC) of Trichophyton species to antifungal agents

Antifungal Agent	Total number of isolates (N)	No. of isolates with MIC (µg/ml)														
		0.0078	0.015	0.03	0.0625	0.125	0.25	0.5	1	2	4	8	16	32	64	128
Miconazole	17	-	-	7	7	3	-	-	-	-	-	-	-	-	-	-
Luliconazole	17	-	-	10	2	2	3	-	-	-	-	-	-	-	-	-
Fluconazole	47	-	-	-	-	-	-	-	-	11	23	10	1	2	-	-
Itraconazole	47	-	-	-	15	27	4	1	-	-	-	-	-	-	-	-
Voriconazole	17	-	-	-	11	2	3	-	-	-	-	-	-	-	-	-
Ketoconazole	47	-	-	-	6	8	19	14	-	-	-	-	-	-	-	-
Clotrimazole	47	-	-	-	-	1	13	31	-	-	-	-	-	-	-	-
Sertaconazole	17	-	-	4	-	-	-	-	-	-	-	-	-	-	-	-
Naftifine	47	-	-	23	7	3	-	-	-	-	-	-	-	-	-	-
Terbinafine	47	-	29	9	-	4	-	-	-	1	2	2	-	-	-	-
Griseofulvin	47	-	-	-	-	-	-	-	-	-	2	-	11	31	-	3
Amorolfine	17	-	10	7	-	-	-	-	-	-	-	-	-	-	-	-
Ciclopirox olamine	47	-	-	-	-	-	47	-	-	-	-	-	-	-	-	-

All isolates showed MIC >1 for fluconazole and griseofulvin. Five isolates showed MIC > 1 for terbinafine and naftifine. MIC of 1 was observed for two sertaconazole in two isolates and voriconazole in one isolate. Molecular detection of terbinafine resistance was done in 15/55 isolates. Two patients had a squalene epoxidase (SE) gene mutation leading to F397L substitutions. The salient features of the patients with dermatophytes with high MIC are given in Table 7.

**Table 7:** Salient features of patients with dermatophytosis with high MIC to azole- and/or allylamine antifungals

Case No.	Age/Sex	Clinical type	Dermatophyte isolated	Risk factor	Previous antifungal exposure	MIC of antifungals (µg/ml)			Mutation
						Terbinafine	Naftifine	Sertaconazole	
1	18/F	T. corporis, cruris	T. mentagrophytes complex	Nil	Unknown	0.0156	0.0312	1	No
2	42/M	T. corporis	T. mentagrophytes complex	Nil	Ketoconazole soap, cream, sertaconazole cream, Miconazole gel, T. fluconazole, T. itraconazole	0.0156	0.0312	1	No
3	50/M	T. unguium	T. mentagrophytes complex	Nil	None	4	4	Not done	No
4	24/M	T. corporis	T. mentagrophytes complex	Family history of infection	Ketoconazole soap, amorolfine cream, clotrimazole cream, C. Itraconazole	4	4	Not done	No
5	21/M	T. corporis	T. mentagrophytes complex	Nil	None	2	4	Not done	No
6	18/M	T. corporis	T. mentagrophytes complex	Nil	Homeopathy	8	8	0.312	F397L
7	62/M	T. corporis	T. mentagrophytes	Nil	Ketoconazole, terbinafine cream, C. Itraconazole od - 1 month, irregular treatment. Then griseofulvin, ketoconazole cream-3 months	8	8	0.5	F397L

Previous therapy with antifungals was given in 32/55 (58.8%) of the patients; other modes of treatment like homeopathy and ayurveda in 4/55 (7.2%). 19/55 (34.5%) of the patients were not treated for the infection. In our institute, the patients were treated with both oral and topical antifungals. The details are given in Table 8.

**Table 8:** Past and present treatment of the patients with dermatophytosis

	Treatment given	No. of patients
Past Treatment	Topical only	6(10.9%)
	Oral and Topical	14(25.4%)
	Only Oral	1(1.8%)
	Treatment taken but unknown	11(20%)
	Homeopathy	2(3.6%)
	Ayurveda	1(1.8%)
	Neem paste	1(1.8%)
	No Treatment	19(34.5%)
Present Treatment	Topical only	4(7.2%)
	Oral and Topical	51(92.7%)

## Discussion

In India, dermatophytosis has emerged as a general public health problem [3]. It affects the patient’s quality of life by restricting social interactions due to the clear visibility of the lesions leading to cosmetic embarrassment [10]. The prevalence of dermatophytosis in India ranges from 36-78% [11]. The present study isolated dermatophytes in 35.4% of the cases. Dermatophytes affect all age groups [2], but most were in the younger age group, from 21 to 40 years, in different studies [5, 8, 9, 12]. There was a male preponderance in some studies [4], possibly due to outdoor work leading to sweating and infections. In some studies, homemakers are involved more due to increased sweating while doing household work. In our study, there is male preponderance.

A study from Telangana found that most patients were active workers (41%), 17% were housewives and 14% were students [5]. In the present study, 19.2% were students and 19.2% were housewives. The rest of the patients were employees in different settings.

In various studies, most patients were from rural areas compared to urban areas [5,13]. In contrast to the previous studies, our data showed that 92% of our patients were from urban areas. In urban populations, many factors, such as lifestyle and place of living, will also differ, leading to the disease.

Patients belonging to lower and middle socioeconomic status were mostly affected due to poor hygienic conditions, overcrowding, and sharing of common items [5,14]. In the present study, 65% of the patients were from middle socioeconomic status.

Several studies across the country reported that family members were affected in 4.5% [5], 20% [15], and 16.6% [14] of cases, and friends in 16% of cases [5] and were the source of infection. Walking barefoot can also lead to infection with dermatophytes [2]. In the present study, family members and roommates of patients were affected, and sharing of combs and towels led to infection in 21.8% of cases.

Tinea corporis is the major clinical type in many studies [9,14,15]. In our study, tinea corporis was the major clinical type in 70.9% of cases.

Many patients have multiple site involvement (34%-77%), and cases presenting with extensive lesions, ring within-ring lesions, multiple steroids-modified lesions, and a chronic course of the disease make the diagnosis and treatment difficult [4, 15, 16]. Multiple-site involvement is mainly associated with factors such as diabetes mellitus, improper treatment, and delay in treatment [15]. In Indian studies, tinea corporis with tinea cruris was the most common type of mixed infection [4, 15]. In the present study, mixed infection is noted in 10 (18.1%) patients, of which seven presented with both tinea corporis and tinea cruris.

Diabetes is one of the important co-morbidity for dermatophytosis [10,17]. The other risk groups include patients on steroid therapy, immunocompromised conditions like HIV, solid organ transplants, etc. These patients are prone to chronic dermatophytosis [2, 18, 19]. In our study, 12.1% of the patients had diabetes and 5.4% were on steroids. Thirty percent of these patients had an infection for more than one-year duration.

If the patient has suffered from the disease for more than six months to one year, with or without recurrence, despite being adequately treated it is referred to as chronic dermatophytosis [11]. Tinea corporis and tinea cruris were the most common clinical forms in patients with chronic lesions [20]. About 60% of our patients had lesions for more than six months duration and of them, 14/33 had been treated adequately. Two of the patients were diabetics, and one of them was on steroids. 26/33 (78.7%) of the patients had tinea corporis, 4/33 (12.1%) had combined infection with tinea corporis and cruris and 3/33 (3%) had tinea unguium. In our study, 7/33 of the patients with chronic dermatophytosis had contact with family members with dermatophytosis and sharing of their items.

There is variation in the prevalence of dermatophyte species from different regions [7, 11]. This may be because dermatophytes are host-specific due to the difference in the composition of keratin in the host [21]. Recently, there has been a shift in the etiology of dermatophytosis from *T. rubrum* to *T. mentagrophytes* [4, 7, 9, 15, 22] and the reason for the change in organism isolated is not clear [9]. In our study, *T. mentagrophytes* complex was the most predominant organism isolated.

Sequencing of the internal transcribed spacer (ITS) region of *T. mentagrophytes* genotype VIII identified them as *T. indotineae* and it was classified as a separate species in 2020 [23]. In a study from India, the multigene sequencing of 219 *Trichophyton* species isolated from human and animal origin was done and all the isolates except one were identified as *T. indotineae* [24]. In the present study, sequencing of the isolates was not done, and was phenotypically identified as *T. mentagrophytes* complex.

Treatment of dermatophytosis involves an oral/topical/multidrug regime (allylamines, triazole, and imidazole group) [25]. An alarming rise in resistance to antifungals leads to increased chronic dermatophytosis cases. Hence there is a need to perform antifungal susceptibility testing of dermatophytes [26]. However, there are no clinical breakpoints for AFST available by standard guidelines [9], and few reports address the correlation between in-vitro resistance and treatment failure. There is no definitive evidence for the same [27].

High MIC for fluconazole was observed in 35.4% [9] and 82.6% [28] of the isolates from studies across India, showing increasing resistance to the drug.

In the present study, all 47 isolates had an MIC of ≥2 µg/ml to fluconazole. Five of our patients had been treated with oral fluconazole before coming to our institute and then were treated with ketoconazole and itraconazole. All the five patients had tinea corporis. Four of them had growth of *T. mentagrophytes* complex in culture and one had growth of *T. rubrum*. Four patients had predisposing risk factors (Visiting a massage center, homeopathy treatment, ayurvedic treatment, steroid treatment). The other patient had a family history of infection and *T. rubrum* was grown in culture. For all the above patients, the MIC of griseofulvin was also high. Other drugs tested showed a low MIC.

About 95.7% of our isolates showed an MIC of ≥16 µg/ml for griseofulvin, similar to other studies [9, 29] with a high MIC of 32.

Terbinafine, a systemic fungicidal drug, inhibits the growth of fungus by inhibiting the squalene epoxidase gene, necessary for ergosterol synthesis. Until recently, resistance and treatment failures to terbinafine have rarely been reported [30]. In a study from India, 20/195 isolates had a high MIC to terbinafine [9]. Resistance to terbinafine is due to the point mutation in the squalene epoxidase gene conferring Leu393Phe and Phe397Leu substitutions, thereby reducing the action of terbinafine [30]. In the present study, 5/47 (10.6%) isolates had MIC of >1 for terbinafine and two of the isolates had F379L mutation in squalene epoxidase gene with terbinafine MIC of 8 µg/ml. One of them is a student with tinea corporis of 18 months duration who took homeopathy treatment. Lesion flared up and he came to our institute. The other patient had tinea corporis of four months duration and was on improper treatment with topical and systemic antifungals. Both of them were treated with Cap. itraconazole along with topical ketoconazole.

Along with drugs such as itraconazole, sertaconazole, ciclopirox olamine, ketoconazole, and naftifine, newer drugs such as amorolfine and luliconazole have lower MIC in vitro and they are shown to have potent fungicidal activity against *Trichophyton* species [29]. All our isolates have lower MIC except for 2/17 isolated which has an MIC of 1 µg/ml for sertaconazole. These patients do not have any significant risk factors. Tinea corporis was the clinical manifestation and *T. mentagrophytes *complex was grown in culture in the patients. One patient had used topical sertaconazole irregularly and in the other patient, treatment was unknown. Both patients were treated with Cap. itraconazole along with topical ketoconazole. Except for the high MIC of fluconazole and griseofulvin, the MIC of other drugs was low in these patients.

Treatment of dermatophytosis includes topical and systemic antifungals. In India, however, fixed drug combination creams with steroid, and antifungal antibacterials are available in the market and irrational and improper usage of them without proper medical advice for years together can lead to treatment failure and chronic dermatophytosis, which is of major concern [3].

Limitations

As it was a retrospective study, follow-up of the patients could not be done. Only phenotypic identification of the isolates was done and the majority of the isolates were identified as *T. mentagrophytes *complex.

## Conclusions

The present study highlights the prevalence, species distribution, and susceptibility pattern of dermatophytes from the southern part of India. *T. mentagrophytes* complex was the predominant species isolated and tinea corporis was the commonest clinical type. Resistance to antifungal drugs like terbinafine, griseofulvin, and fluconazole has been noted. The possibility of chronicity/recurrence of the lesions is also high. Hence, dermatophytosis has become a difficult-to-treat disease, and therefore, utmost care should be taken to provide optimal treatment for the patients in selecting the proper drug, dosage and duration of therapy. Also, education of the patients regarding personal hygiene and adherence to treatment protocol can prevent recalcitrant lesions.
